# Radiotherapy–immunotherapy combinations – perspectives and challenges

**DOI:** 10.1002/1878-0261.12658

**Published:** 2020-03-13

**Authors:** Michele Mondini, Antonin Levy, Lydia Meziani, Fabien Milliat, Eric Deutsch

**Affiliations:** ^1^ Gustave Roussy Université Paris‐Saclay SIRIC SOCRATE Villejuif France; ^2^ INSERM U1030 Labex LERMIT Villejuif France; ^3^ Département de Radiothérapie Gustave Roussy Université Paris‐Saclay DHU TORINO Villejuif France; ^4^ Department of Radiobiology and regenerative Medicine (SERAMED) Laboratory of Medical Radiobiology (LRMed) Institute for Radiological Protection and Nuclear Safety (IRSN) Fontenay‐aux‐Roses France

**Keywords:** abscopal effect, immune modulation, immunotherapy, radiotherapy, toxicity

## Abstract

Ionizing radiation has historically been used to treat cancer by killing tumour cells, in particular by inducing DNA damage. This view of radiotherapy (RT) as a simple cytotoxic agent has dramatically changed in recent years, and it is now widely accepted that RT can deeply reshape the tumour environment by modulating the immune response. Such evidence gives a strong rationale for the use of immunomodulators to boost the therapeutic value of RT, introducing the era of ‘immunoradiotherapy’. The increasing amount of preclinical and clinical data concerning the combination of RT with immunomodulators, in particular with immune checkpoint inhibitors such as anti‐PD‐1/PD‐L1 and anti‐CTLA4, reflects the interest of the scientific and medical community concerning immunoradiotherapy. The expectations are enormous since the rationale for performing such combinations is strong, with the possibility to use a local treatment such as RT to amplify a systemic antitumour response, as illustrated by the case of the abscopal effect. Nevertheless, several points remain to be addressed such as the need to find biomarkers to identify patients who will benefit from immunoradiotherapy, the identification of the best sequences/schedules for combination with immunomodulators and mechanisms to overcome resistance. Additionally, the effects of immunoradiotherapy on healthy tissues and related toxicity remain largely unexplored. To answer these critical questions and make immunoradiotherapy keep its promising qualities, large efforts are needed from both the pharmaceutical industry and academic/governmental research. Moreover, because of the work of both these entities, the arsenal of available immunomodulators is quickly expanding, thus opening the field to increasing combinations with RT. We thus forecast that the field of immunoradiotherapy will further expand in the coming years, and it needs to be supported by appropriate investment plans.

## Graphical abstract

This review briefly summarizes the results obtained so far using the combination of immunomodulators with radiotherapy (immunoradiotherapy), both preclinically and clinically, and discusses the perspectives and challenges, including the still limited exploration of the effects of immunoradiotherapy on healthy tissues and related toxicity.

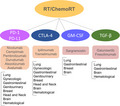

**Abbreviations**
cGAS: cyclic GMP‐AMP synthase
CTLA4: cytotoxic T‐lymphocyte‐associated protein 4
DAMPs: damage‐associated molecular patterns
DCs: dendritic cells
GM‐CSF: granulocyte‐macrophage colony‐stimulating factor
HMGB1: high‐mobility group box 1
ICD: immunogenic cell death
ICI: immune checkpoint inhibitors
IFNβ: interferon beta
IR: irradiation
IRF3: interferon regulatory factor 3
LAG‐3: lymphocyte‐activation gene 3
MDSC: myeloid‐derived suppressor cells
PD‐1: programmed cell death protein 1
PD‐L1: programmed death‐ligand 1
RT: radiotherapy
SBRT: stereotactic body radiation therapy
STING: stimulator of interferon genes
TAM: tumour‐associated macrophages
TGF‐β: transforming growth factor beta
TIM‐3: T‐cell immunoglobulin and mucin‐domain containing‐3
Treg: regulatory T cells

## 1 Introduction

The efficacy of radiotherapy (RT) has largely improved in recent decades, mainly due to improved treatment planning, imaging and novel irradiation (IR) techniques. On the other hand, the promise to pharmacologically improve the therapeutic index of RT, that is, by combining it with drugs such as DNA repair inhibitors, pro‐apoptotic agents or antiangiogenic agents has been broken by unexpected and deceiving results (Chargari *et al.*, 2016). This scenario has largely changed in recent years, and new hopes relying on a combination of RT with drugs have transpired since the ‘immunotherapy revolution’ in oncology has begun. Indeed, a ‘paradigm shift’ has been proposed when a large bulk of experimental data provided enough evidence that some of the effects of ionizing radiation contribute to antitumour immunity (Formenti and Demaria, 2013). Currently, it is widely accepted that RT does not act merely as a cytotoxic agent, but it can deeply reshape the tumour environment in many ways and in particular by modulating the immune response (Frey *et al.*, 2017), giving a strong rationale to use immunomodulators to boost the therapeutic value of RT (immunoradiotherapy).

Herein, we briefly summarize the results obtained so far using immunoradiotherapy, both preclinically and clinically, and we discuss the perspectives and challenges, including the still limited exploration of the effect of immunoradiotherapy on healthy tissues.

## 2 Rationale for immunoradiotherapy combinations

### 2.1 Immunostimulation

Historically, the main rationale for the use of ionizing radiation to treat cancer initially came from empirical clinical observations and has been attributed to its cytotoxic activity, in particular by inducing DNA damage eventually leading to tumour cell killing. Consequently, most efforts in classical radiobiology have been spent trying to improve tumour cell killing by IR and/or to reduce the damage to healthy cells, that is, to improve the differential cytotoxic effect of RT (Bhattacharya and Asaithamby, 2017). Even though some effects of RT on the immune system have been known since the late 1970s (Stone *et al.*, 1979), the view of cancer as a mostly cell‐autonomous process together with preclinical research on RT performed using *in vitro* models and *in vivo* models in immunodeficient mice did not allow researchers to fully appreciate the contribution of the immune response to the therapeutic effect. When the landscape of oncology research shifted towards a view of cancer as disease strongly affected by the tumour stroma, radiation biology started to accumulate evidence of the involvement of the immune system in mediating its therapeutic efficacy, especially in the last decade.

Ionizing radiation has been recognized as one of the anticancer agents able to induce ‘immunogenic cell death’ (ICD), a type of cell death that promotes a T‐cell‐mediated immune response against antigens derived from dying cells (reviewed in Wennerberg *et al.*, 2017). Ionizing radiation has the potential to activate the key molecular steps (e.g., the translocation of the ER protein calreticulin to the cell surface, the release of the nuclear protein HMGB1 and the release of adenosine triphosphate) described as ‘damage‐associated molecular patterns’ (DAMPs) and that are required to achieve ICD. This process enhances the uptake of tumour‐derived antigens, including neo‐antigens due to immunogenic mutations driven by RT, by antigen‐presenting cells such as dendritic cells (DCs) and macrophages. This action is accompanied by the release of pro‐inflammatory cytokines, such as CXCL10 and CXCL16, which, together with the RT‐promoted process of vascular normalization (Klug *et al.*, 2013; Mondini *et al.*, 2015), can enhance the infiltration of activated CD8^+^ T cells (Matsumura *et al.*, 2008). It has also recently been reported that newly recruited T cells contribute to RT efficacy and that tumour‐reprogrammed tissue‐resident T cells, which can survive to clinically used RT doses, can mediate tumour control (Arina *et al.*, 2019).

Another central element contributing to immunostimulation by RT is the induction of the interferon (IFN) cascade through the activation of the STING DNA‐sensing pathway. After exposure to IR, DNA can shuttle to the cytoplasm and be sensed by cGAS, which triggers the nuclear translocation of STING. Through a signalling cascade that leads to the activation of IRF3, STING leads to the production of type I interferon (Galluzzi *et al.*, 2018), fostering the maturation of DCs and their antigen presentation to T cells, contributing to the amplification of an antitumour adaptive immune response. It interesting to note that the secretion of type I IFN appears to be dependent on the irradiation dose, where 8 Gy fractions induce a strong upregulation of IFNβ levels, while higher doses failed to achieve this effect.

In addition, RT has been shown to enhance the expression of major histocompatibility complex‐I molecules favouring antigen presentation (Reits *et al.*, 2006). IR doses in the range of those most used in clinical settings (2 Gy) have also been shown to reprogram the phenotype of tumour‐associated macrophages (TAMs) towards pro‐immunogenic behaviour (Klug *et al.*, 2013).

All these observations demonstrate that RT can trigger both the adaptive and innate immune responses towards antitumour activity, thus enhancing immunotherapy.

### 2.2 Abscopal effect

The occurrence of tumour responses at sites distant from the irradiated volume has been known for more than 60 years and is termed the abscopal effect (Brix *et al.*, 2017). The abscopal effect is a rare event, and the number of cases described in the literature is extremely low. Indeed, only 47 cases were reported from 1960 to 2018 in patients treated with RT alone. With the advent of the immunoradiotherapy era, this number is quickly growing, with the same number of cases (47) described in 6 years (2012–2018) in patients treated with immunomodulators combined with RT compared to 50 years for RT‐only treated patients (Dagoglu *et al.*, 2019). This finding likely results from the induction of a systemic immune response trigger by the combined immunostimulatory effect of RT with immunotherapy. Although investigated in preclinical settings, the precise mechanisms underlying abscopal responses remain unclear and relatively unexplored in clinical settings (Rodriguez‐Ruiz *et al.*, 2019), even if the first clues stem from dedicated clinical trials (Formenti *et al.*, 2018; Golden *et al.*, 2015), in which immunoradiotherapy resulted in 18–27% of abscopal responses.

One of the most ambitious goals of immunoradiotherapy is to make the occurrence of the abscopal response systematic. In addition, the abscopal effect is a direct witness of the systemic synergy of immunoradiotherapy that can also be exploited at the locally advanced tumour stage to prevent metastasis and increase control, as illustrated by the recent approval of immune checkpoints inhibitors (ICI) adjuvant to chemoradiation in locally advanced lung cancer. A large amount of investigations is still needed to understand why abscopal effect remains a relatively rare phenomenon, with its clinical benefit restricted to a proportion of patients. One explanation is that the immunosuppressive mechanisms amplified by RT could limit a widespread systemic immune response.

### 2.3 Immunosuppression

As the balance between stimulating and suppressive signals finely tunes the activity of the immune system, it is not surprising that together with immunostimulatory responses, RT can also trigger immunosuppression. For example, RT has been shown to upregulate the expression of the immune checkpoint PD‐L1 in both preclinical (Deng *et al.*, 2014) and clinical settings (Twyman‐Saint Victor *et al.*, 2015), thus limiting the activation of tumour T cells. RT can also enhance the release of immunosuppressive cytokines such as transforming growth factor beta (TGF‐β) in the tumour environment. TGF‐β can repress the proliferation, activation and effector function of T cells and can also impact the maturation and function of tumour natural killer cells and macrophages; on the other hand, TGF‐β promotes Treg differentiation (Dahmani and Delisle, 2018). Tregs can contribute to immunosuppression after RT, as their infiltration is increased in irradiated tumours (Mondini *et al.*, 2019; Muroyama *et al.*, 2017). RT also triggers an influx of myeloid cells through the upregulation of the secretion of chemokines such as CCL2 by tumours, which can contribute to the generation of an immunosuppressive environment (Kalbasi *et al.*, 2017; Mondini *et al.*, 2019).

The interplay between these populations and inhibitory signals can thus impair or limit an effective antitumour immune response after irradiation.

### 2.4 Combination of radiotherapy with immunotherapeutics

Given the essential role of lymphocytes in the response to RT, the combination of RT with immune checkpoint inhibitors (ICI) can unleash the potential of the T‐cell compartment. Associations of anti‐CTLA4 with RT were among the first immunoradiotherapy combinations tested, which gave promising results such as the induction of systemic immune responses. As PD‐L1 expression is increased after RT, the combination of RT with anti‐PD‐1/PD‐L1 has a strong rationale, and it has been widely tested in preclinical settings and then translated into clinical trials (Shevtsov *et al.*, 2019).

Even if the combination of RT and anti‐CTLA4 or PD‐1/PD‐L1 can ameliorate the efficacy of RT, several mechanisms of resistance to therapies can be developed by the tumours. Thus, many additional immunomodulators are being tested in combination with RT (either alone or together with anti‐CTLA4 and anti‐PD‐1/PD‐L1) such as ICI including anti‐TIM‐3 or anti‐LAG‐3. Other strategies for novel immunoradiotherapies include the administration of costimulatory molecules as agonists of OX‐40 or CD‐40. The modulation of the tumour environment also has a strong rationale, such as targeting the TGF‐β pathway or chemo‐attractive axes such as that of CCL2/CCR2. Other new treatment approaches are based on the use of agents aimed at increasing antigen presentation as agonists of Toll‐like receptors or at boosting the RT‐induced interferon response using modulators of the cGAS/STING pathway.

The arsenal of available immunomodulators is rapidly expanding, paving the way for novel potent immunoradiotherapies but also unravelling several new challenges, such as identifying the optimal combination of molecules, the sequence of administration and the handling of potential associated toxicities.

## 3 Immunoradiotherapy and normal tissue toxicity

The enthusiasm for immunotherapy and radiation therapy combinations also raises the question of normal tissue toxicity and the safety of these treatments (Deutsch *et al.*, 2019). For radiation therapy alone, normal tissue injuries are the main limiting factor of the dose that could be delivered to the target. For organs at risk, tolerance doses and volumes are defined according to clinical treatment guidelines and are based on several parameters including the fractionation of the dose and the organization of the organ at risk. Moreover, concerning immunotherapy alone, previous clinical reports have described acute and long‐term toxicities to the skin, colon, liver and lungs (Michot *et al.*, 2016). For example, lung cancer will increasingly be treated using stereotactic body radiation therapy (SBRT), and several clinical trials plan to combine SBRT and ICI (Lin *et al.*, 2019). Moreover, it was reported that patients treated with concomitant ICI and SBRT can develop radiation pneumonitis even if it remains unclear whether the pneumonitis is due to SBRT or to an enhancing effect of the combination with ICI (Delaunay *et al.*, 2017; Louvel *et al.*, 2018). Preclinical experiments using small animals are very useful in translational cancer research and the clinical implementation of novel treatments. The influence of dose fractionation has not yet been satisfactorily investigated in depth. Preclinical models are now available to model lung SBRT in rodents, and the method to deliver the dose (in terms of fraction) associated with immunotherapy could be used to obtain crucial information about putative acute and long‐term toxicity (Lavigne *et al.*, 2019). Moreover, follow‐up clinical data are not yet available about the effects of SBRT/immunotherapy combination, and prospective data from a large series of patients regarding safety are a key issue for the future.

Radiation‐induced normal tissue injuries are characterized by a very complex dynamic process involving a large number of molecular and cellular factors. The immune system is known to play a pivotal role not only in the onset of cancer development, but also in radiation‐induced normal tissue injury. There is a close association between inflammation and injury, and innate immune cells, including neutrophils, monocytes and macrophages, are the first line of defence against infection and release highly toxic chemicals to kill pathogens. Most of these molecules act as DAMPs. However, similar mediators are also released from immune cells for the resolution of injury and the proper wound healing process. The outcome (toxicity or not) is dependent on many parameters, including the phenotype and molecular footprints of these cells, their relative spatial localization and the dynamics of their recruitment/elimination. It is crucial to obtain more information about the effect of immunotherapy/RT combinations on the temporal and spatial effects on immune cells, which are involved in normal tissue toxicity. For example, it was shown that TAMs express PD‐1 and that immunotherapy could also act through a direct effect on macrophages (Gordon *et al.*, 2017). However, macrophages play a crucial role not only in the development of inflammation but also in its resolution as well as in tissue regeneration. Details are still lacking about what happens to macrophage subpopulations in normal tissues exposed to immunoradiotherapy and the consequences of their putative toxic effects (Meziani *et al.*, 2018). From a more general point of view, this issue raises the question about the relative contribution and biologic significance of PD‐1/PD‐L1 expression by cells in normal tissue and how such expression could be involved in response to immunoradiotherapy combination regimens. Pertinent preclinical models combined with state‐of‐the‐art methods of molecular biology such as ‘single‐cell RNA‐seq’ could help answer several concerns and notably to exactly identify the effects of RT/immunotherapy combinations on both pro‐regenerating cell types and cell types involved in the toxic effects.

Experiments to obtain information on the toxicity of normal tissues will be very important for translation into clinical applications. In general, these concerns are not priorities compared to research projects aimed at investigating the antitumour effects of combination treatments. However, the recent history of combinations of targeted therapies with radiotherapy (such anti‐antigenic factors) has taught us that this aspect should not be overlooked.

## 4 Immunoradiotherapy in the clinical tracks

**Fig. 1:**
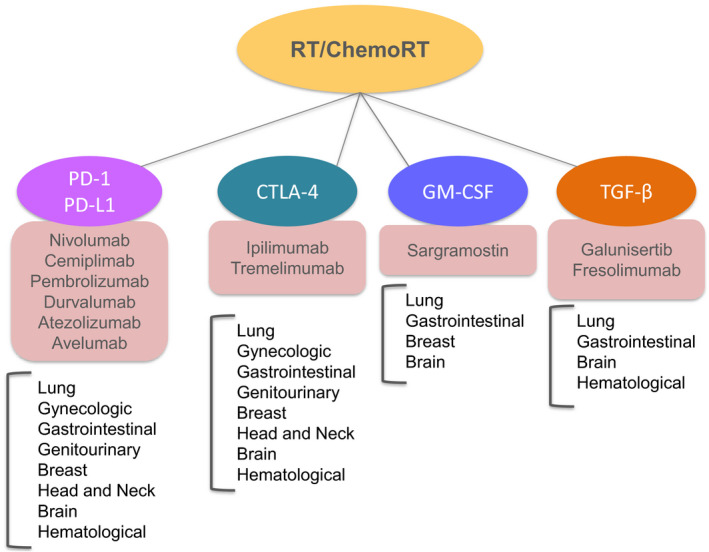
Immunomodulators used in ongoing/past clinical trials, in combination with radiotherapy/chemoradiotherapy. For each immunotherapy category, main drug names and the tumour sites investigated are listed.

Immunotherapies using anti‐PD‐L1 and/or anti‐CTLA4 have recently become new standards of care in several cancer types. Given the strong abovementioned rationale for combining ICIs with radiotherapy, there are a rapidly increasing number of immunoradiotherapy clinical trials (Fig. 1; Cushman *et al.*, 2018; Kang *et al.*, 2016). The first clinical advantage of triggering a systemic (out of the radiation field, ‘abscopal’), immune response was presented in a melanoma patient progressing on ipilimumab (anti‐CTLA4, Postow *et al.*, 2012). A secondary analysis of a phase I study assessing the anti‐PD‐1 agent pembrolizumab in patients with advanced non‐small‐cell lung cancer (NSCLC) then showed that patients who received radiotherapy before (median: 9 months) the anti‐PD1 treatment had increased progression‐free survival (PFS) and overall survival (OS) compared with patients who did not receive prior radiotherapy (Shaverdian *et al.*, 2017). One of the largest randomized clinical trials (CA184‐043 study) on advanced castration‐resistant prostate cancer receiving palliative (single fraction of 8 Gy) radiotherapy on a bony lesion compared the anti‐CTLA4 agent ipilimumab versus placebo. The trial was negative for OS, but it should be noted that ipilimumab alone (without irradiation) did not become a standard in such patients (Kwon *et al.*, 2014). The recent PACIFIC trial assessing the anti‐PD‐L1 agent durvalumab as consolidation after thoracic chemoradiotherapy in NSCLC patients demonstrated an improved OS compared to placebo (Antonia *et al.*, 2018). In a multicentre, randomized phase 2 study (PEMBRO‐RT) of 92 patients with advanced NSCLC, better outcomes were observed when SBRT (3 times 8 Gy) was administered 7 days before the anti‐PD1 agent pembrolizumab compared to the nonirradiated group (Theelen *et al.*, 2019). Since then, several prospective trials have assessed the addition of a PD‐1/PD‐L1 to concurrent chemoradiotherapy (e.g., NCT03728556, NCT03519971, NCT03745222: RATIONALE001) or to SBRT (e.g., NCT03774732: NIRVANA‐Lung) in NSCLC patients.

The results from the first clinical trials, though encouraging, have shown that most patients do not respond to immunoradiotherapy combinations. Indeed, several limitations should be highlighted. (a) The best surrogate immune biomarker for radioimmunotherapy remains to be confirmed. In an unplanned post hoc subgroup analysis based on PD‐L1 expression levels on tumour cells at initial biopsy, a benefit in OS was not proven in the PD‐L1‐negative patients of the PACIFIC trial (Antonia *et al.*, 2018). In contrast, positive results were largely influenced by the PD‐L1‐negative subgroup in the PEMBRO‐RT trial (Theelen *et al.*, 2019). Further potential predictive and prognostic immune assays are being studied at the cellular (tumour microenvironment composition), genomic (mutational/neoantigen load) and peripheral (blood and microbiota) levels (Levy *et al.*, 2017). Radiomics approaches, using artificial intelligence algorithms to analyse radiographic characteristics, have been recently proposed as noninvasive biomarkers for response to immunotherapy and may be useful to improve patient stratification and for predicting clinical outcomes of patients treated with immunotherapy (Sun *et al.*, 2018; Trebeschi *et al.*, 2019). (b) The best volume of irradiation in patients receiving ICI may be different from that in patients without receiving immunostimulatory agents, in particular concerning the need to perform elective lymph node irradiation (Luke *et al.*, 2018). Irradiation in great vessels and draining lymph nodes (main location of T‐cell cross‐priming by DCs) could affect immune cell functions and migration (Deutsch *et al.*, 2019). Modern techniques such as volumetric modulated arc therapy (VMAT) induce larger volumes of healthy tissues receiving low doses of ionizing radiation that could affect circulating lymphocytes and decrease the adaptive immune response (Tang *et al.*, 2014). (c) The immune response may depend on the timing, the number of irradiated sites and the employed dose fractionation. The irradiation of many lesions within multiple tissue beds could increase the repertoire of released antigens and activate immune signals from various tumour microenvironments (Brooks and Chang, 2019). For instance, in the CA184‐043 trial, a single dose fraction of 8 Gy to the bone did not lead to strong immune stimulation (Kwon *et al.*, 2014), while multiple fractions (at least 3 sessions) of ‘moderate’ dose level to the lung (cf. PEMBRO‐RT study) have been more efficient in stimulating the immune response (Theelen *et al.*, 2019). (d) Negative effects (radiation‐induced toxicity and immune‐related events) could also be increased through the synergistic stimulation of both local and systemic immunities. Ideally, the pretreatment determination of individuals’ normal tissue and/or tumour sensitivity will help identify patients more likely to achieve the best therapeutic index ratio (Chargari *et al.*, 2016). (e) Abscopal response, as it has not been sufficiently well documented. Stratification of patients and monitoring of out‐of‐field responses are, however, now incorporated in current trials (e.g. NCT03426657 and NCT03386357 for head and neck cancers).

## 5 Conclusions

**Fig. 2:**
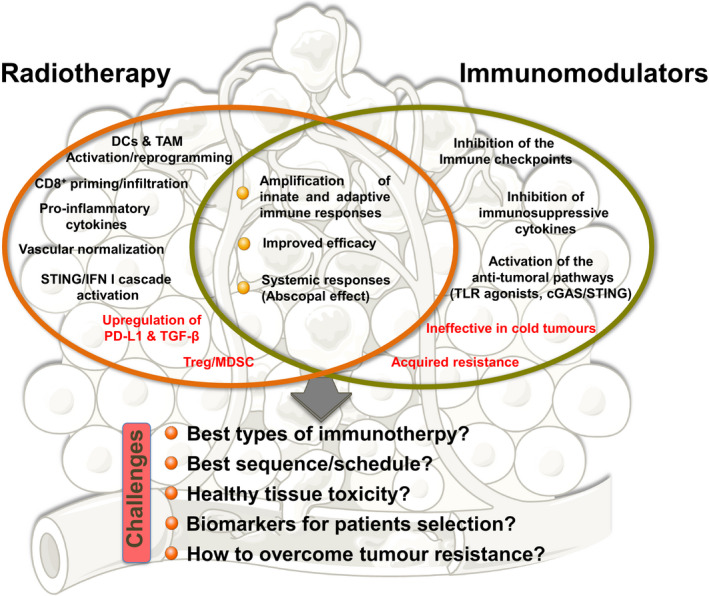
Strengths, weaknesses and challenges of radiotherapy and immunotherapy combinations.

The large and increasing amount of preclinical and clinical data concerning the combination of immunomodulators with radiotherapy reflects the great interest of the scientific and medical community concerning immunoradiotherapy. The expectations are enormous since the rationale for performing such combinations is strong, and the first positive results are coming from clinical settings. Nevertheless, several points remain to be addressed or better elucidated (Fig. 2) such as (a) what are the best sequences/schedules to follow when combining RT with immunomodulators; (b) how to select the best types of immunotherapy; (c) what are the effects of immunotherapies on healthy tissues; (d) which biomarkers are useful for selecting the best candidates for immunoradiotherapies; (e) how to overcome resistance and increase the number of responsive patients; and (f) how to increase the (still very limited) number of systemic antitumour responses leading to an effective abscopal effect.

To answer these critical questions and make immunoradiotherapy keep its promising qualities, great efforts are needed from both the pharmaceutical industry and academic/governmental research. Moreover, because of the work of both these entities, the arsenal of available immunomodulators is quickly expanding, thus providing the field with increasing combinations with RT. We thus forecast that the field of immunoradiotherapy will further expand in the coming years, and it needs to be supported by appropriate investment plans.

## Conflict of interest

ED, MM and LM declare grants from Roche Genentech, Servier, AstraZeneca, Merck Serono, Bristol‐Myers Squibb, Boehringer Ingelheim, Eli Lilly and MSD, outside the submitted work. ED declares personal fees from Roche Genentech, AstraZeneca, MSD, AMGEN, Accuray and Boehringer Ingelheim outside the submitted work. ED declares shared patents with NH‐Theraguix and Clevexel.
